# Chemical Characterization and Beneficial Effects of Walnut Oil on a *Drosophila melanogaster* Model of Parkinson’s Disease

**DOI:** 10.3390/molecules29174190

**Published:** 2024-09-04

**Authors:** Rossella Avallone, Cecilia Rustichelli, Monica Filaferro, Giovanni Vitale

**Affiliations:** 1 Department of Life Sciences, University of Modena and Reggio Emilia, 41125 Modena, Italy; rossella.avallone@unimore.it; 2 Department of Biomedical, Metabolic and Neural Sciences, University of Modena and Reggio Emilia, 41125 Modena, Italy; monica.filaferro@unimore.it

**Keywords:** walnut oil, omega-3 fatty acids, α-linolenic acid, neurodegeneration, Parkinson disease, *Drosophila melanogaster*, dopamine

## Abstract

A nutritional approach could be a promising strategy to prevent or decrease the progression of neurodegenerative disorders such as Parkinson’s disease (PD). The neuroprotective role of walnut oil (WO) was investigated in *Drosophila melanogaster* treated with rotenone (Rot), as a PD model, WO, or their combination, and compared to controls. WO reduced mortality and improved locomotor activity impairment after 3 and 7 days, induced by Rot. LC-MS analyses of fatty acid levels in *Drosophila* heads showed a significant increase in linolenic (ALA) and linoleic acid (LA) both in flies fed with the WO-enriched diet and in those treated with the association of WO with Rot. Flies supplemented with the WO diet showed an increase in brain dopamine (DA) level, while Rot treatment significantly depleted dopamine content; conversely, the association of Rot with WO did not modify DA content compared to controls. The greater intake of ALA and LA in the enriched diet enhanced their levels in *Drosophila* brain, suggesting a neuroprotective role of polyunsaturated fatty acids against Rot-induced neurotoxicity. The involvement of the dopaminergic system in the improvement of behavioral and biochemical parameters in *Drosophila* fed with WO is also suggested.

## 1. Introduction

Growing scientific evidence supports the potential efficacy of polyunsaturated fatty acid (PUFA) supplementation in neurodegenerative disorders such as Parkinson’s disease (PD) and Alzheimer’s disease (AD) [1]. While dietary recommendations cannot cure PD or AD, they may help alleviate some symptoms and slow the cognitive and physical decline associated with these conditions.

The main classes of PUFAs include omega-3, which comprises α-linolenic acid (ALA, 18:3 ω-3), eicosapentaenoic acid (EPA, 20:5 ω-3), and docosahexaenoic acid (DHA, 22:6 ω-3), and omega-6, which includes linoleic acid (LA, 18:2 ω-6) and arachidonic acid (ARA, 20:4 ω-6).

Humans can synthesize saturated and monounsaturated fatty acids (MUFAs) but lack the enzyme omega-3-desaturase required to synthesize ALA and LA [2]. LA and ALA compete for the same conversion enzymes, leading to competitive inhibition between the two substrates. A high intake of LA can shift the balance towards the conversion of omega-6 PUFAs, thereby inhibiting the conversion of ALA to DHA [3].

The PUFA content of membrane phospholipids is crucial for maintaining optimal membrane properties, influencing the activity of membrane-associated proteins and the dynamic functions of membranes. Additionally, free PUFAs are key regulators of gene expression. In mammals, PUFAs have been identified as ligands for several nuclear hormone receptors (NHRs), such as peroxisome proliferator-activated receptors (PPARs) and liver X receptors (LXRs). PUFAs also influence the activity of the transcription factor sterol regulatory element binding protein (SREBP) [4]. Recently, a series of G-protein coupled receptors activated by specific PUFAs were discovered in mammals [5].

Since omega-3 and omega-6 fatty acids are essential, they must be obtained through the diet. The World Health Organization (WHO) currently recommends a dietary omega-6/omega-3 ratio between 1/1 and 4/1. However, in most developed and developing countries, the actual dietary intake exceeds a ratio of 10/1 [6] due to the high amounts of omega-6, particularly ARA, present in everyday diets and fast foods. An imbalanced omega-6/omega-3 ratio negatively impacts the brain’s structure and function, cellular integrity, and the immune system’s physiological status [7]. Diets high in ARA can promote the development of various diseases, while omega-3 PUFAs can positively influence neuronal transmission by altering phospholipid composition and enhancing the fluidity of central nervous system (CNS) cell membranes. This highlights the strong link between PUFA levels and brain functions like neurotransmission and behavior, with diets low in omega-3 PUFAs being detrimental to health.

Considering the potential benefits of PUFA supplementation in neurodegenerative disorders [8], omega-3 fatty acids are among the most prescribed supplements, with their usage predicted to increase by 6.5% from 2023 to 2032. The market for these supplements is expected to exceed USD 4.5 billion by 2032 [8]. Since omega-3 fatty acids are supplements that do not require testing or approval by official regulatory agencies, their role in routine clinical practice needs further evidence to be established.

Various sources of long-chain PUFAs include both aquatic and animal origins. Traditionally, fish and krill oil have been the primary sources of omega-3 fatty acids, including EPA and DHA [9]. However, this practice contributes to the overexploitation of oceans and unsustainable fishing practices. Animal sources such as beef, lamb, pork, poultry, and dairy products contain fatty acids influenced by the animals’ diets and digestive systems. Meat muscle and adipose tissue are rich in ALA, EPA, and DHA, and egg yolks also have high DHA concentrations (around 0.7%), which can increase when chickens’ diets are supplemented with fish oils. Key plant sources of ALA include black raspberry seed oil, with a concentration of 35%, as well as cranberry, basil seed oil, chia seed oil, flaxseed, and walnut oil [10].

Walnut oil (WO) is derived from the kernels of walnut tree fruits (*Juglans regia* L.). The primary active components in WO include fatty acids, phenols, sterols, and minerals, all of which contribute to its significant health benefits [11]. Several studies have demonstrated the antioxidant properties of WO, attributing these effects to its polyphenol content [12]. Additionally, WO has been shown to possess anti-inflammatory, antitumor, immunomodulatory, neuroprotective, and cardioprotective properties [13,14,15,16,17]. It is also utilized in the treatment of diabetes [18] and hyperlipidaemia [19].

The causes of Parkinson’s disease (PD) remain uncertain to this day. However, some researchers have shown that in the brain, excessive production of reactive oxygen species (ROS) or deficiencies and/or weaknesses in antioxidant systems can lead to oxidative stress, which is responsible for neuronal damage and subsequent deterioration [20]. To study the etiology and underlying mechanisms of PD in the search for new therapeutic strategies, researchers have developed both in vitro and in vivo models that replicate the disease’s key characteristics, particularly the degeneration of dopaminergic neurons [21]. Currently, the most widely used experimental models are those based on neurotoxins, which are compounds capable of selectively inducing the death of dopaminergic neurons.

Since the early 20th century, the fruit fly, *Drosophila melanogaster*, has been a key model organism in experimental research for several reasons. First, each fertilized female produces a large number of offspring, and the life cycle is short, with the developmental time from egg to adult being about nine to ten days at 25 °C [22]. This allows researchers to quickly obtain a sufficient number of flies for experiments. Second, *Drosophila* can be raised and maintained in large numbers at a moderate cost. Additionally, their relatively short lifespan, with males living up to approximately 60 days and females up to 80 days depending on conditions and strains, facilitates lifelong studies. Another significant advantage is that the *Drosophila* genome has been fully sequenced [23], and nearly 75% of human disease-related genes have functional orthologs in the fruit fly [24]. According to some reports, *Drosophila melanogaster* possesses a body full of adipose tissue and a lipid transport system similar to that of humans [25]. The fly treatments with herbicides such as paraquat, or with insecticides such as rotenone (Rot), allows researchers to observe phenomena such as oxidative stress, degeneration of dopaminergic neurons, and locomotor defects [26], making it an in vivo useful model for studying neurodegeneration in PD [21,27,28,29]. In recent years, the value of *Drosophila* in nutrition research has gained recognition. Researchers have investigated the effects of different dietary compounds, diet compositions, and plant bioactive components on various health outcomes [30]. Typically, a standard diet for *Drosophila* includes a complex medium of yeast, sugar, and agar, which is easy to prepare and provides all necessary nutrients [31]. However, *Drosophila* does not possess homologs to the Δ5 and Δ6 desaturases and thus cannot synthesize the 20–22 carbon PUFAs from essential fatty acid precursors [25]. As a result, flies must acquire PUFAs from their vegetarian diet, which is primarily restricted to the essential 18-carbon fatty acids LA and ALA.

This study aimed to determine the neuroprotective role of WO in *Drosophila* treated with the toxin Rot as an in vivo model of PD. Rot interferes with mitochondrial respiration by specifically inhibiting complex I of the electron transport chain, leading to ATP depletion, the release of oxygen free radicals, and the loss of the mitochondrial membrane potential. Fatty acid and dopamine (DA) levels in fly heads were determined in *Drosophila* after treatment with WO and Rot, alone or in combination with WO, compared to flies fed with a control diet; locomotor activity and longevity assay were also evaluated. The WO-enriched diet was tested to a) assess its capacity to increase *Drosophila* brain PUFA levels and DA content, and b) study its protective effect in the *Drosophila melanogaster* model of chemically induced PD.

## 2. Results and Discussion

### 2.1. Quantitative Analysis of Fatty Acids in Walnut Oil, Drosophila Food, and Yeast

**Figure 1:**
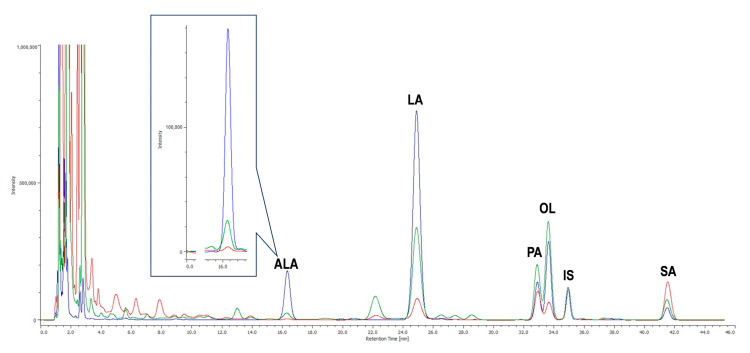
Fatty acid analysis in food samples: representative HPLC-UV chromatograms of walnut oil (WO, blue trace), *Drosophila* food (red trace), and yeast (green trace); ALA: linolenic acid, LA: linoleic acid, PA: palmitic acid; OL: oleic acid, IS: internal standard, SA: stearic acid.

WO was analyzed by HPLC-UV (Figure 1) after saponification and derivatization of the triglyceride fraction, which accounts for 99% of the total lipids present. 

WO was found to contain a high level of LA (594.3 ± 16.13 μg/mg WO), followed by oleic acid (OL, 162.5 ± 3.17 μg/mg WO), ALA (107.5 ± 3.68 μg/mg WO), and then by the saturated palmitic acid (PA, 68.84 ± 1.11 μg/mg WO) and stearic acid (SA, 26.71 ± 0.99 μg/mg WO). These data, expressed as the mean ± standard error of the mean (SEM) from three independent experiments, correspond to percentage values of 59.43 ± 1.61% (LA), 16.25 ± 0.32% (OL), 10.75 ± 0.37% (ALA), 6.88 ± 0.11% (PA), and 2.67 ± 0.10% (SA). The observed fatty acid composition (9.6% SFA-saturated, 16.3% MUFA-monounsaturated; 70.2% PUFA-polyunsaturated) is consistent with the reported nominal composition of WO (9.1% SFA, 16.5% MUFA; 69.9% PUFA). 

*Drosophila* food and yeast were analyzed by HPLC-UV (Figure 1) after lipid extraction (2% and 7%, respectively), triglyceride saponification, and derivatization, as described in the Section 3. The two most abundant fatty acids in *Drosophila* food were LA (4.61 ± 0.08) and SA (4.76 ± 0.44). The mean levels of PA and OL were 2.99 ± 0.21 and 2.67 ± 0.07, respectively, while ALA (0.13 ± 0.006) was present at a significantly lower level (*p* < 0.0001) compared to the other fatty acids (data expressed as μg/mg food ± SEM from three independent experiments). The predominant fatty acid in yeast was LA (14.64 ± 0.55), followed by OL (10.95 ± 0.45), PA (5.44 ± 0.31) and SA (2.14 ± 0.33). The amount of ALA (0.98 ± 0.08) was significantly lower (*p* < 0.0001) than that of the other fatty acids (data expressed as μg/mg yeast ± SEM from three independent experiments). EPA, DHA, and ARA were not detected in WO, *Drosophila* food, or yeast.

**Figure 2:**
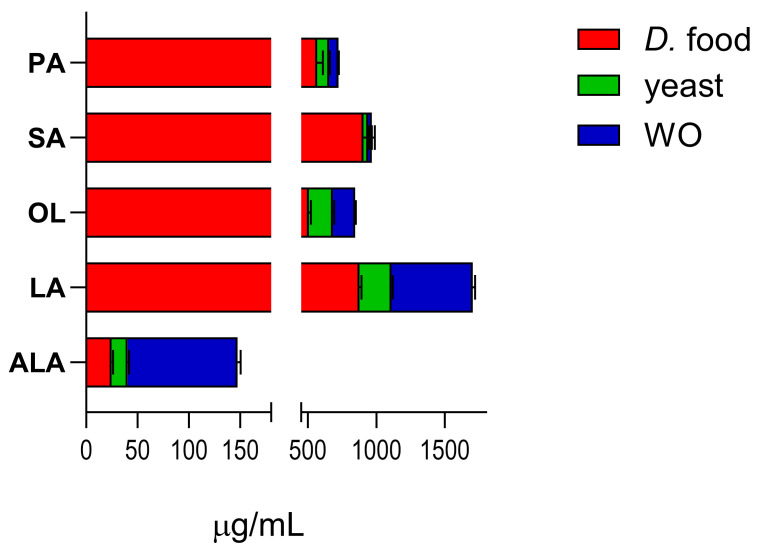
Fatty acid content in *Drosophila* food, yeast, and WO. Data are expressed as μg/mL diet ± SEM (three independent experiments).

Figure 2 shows the content of the target fatty acids in the standard diet (*Drosophila* food and yeast) and in diet enriched with WO.

Enrichment of the standard *Drosophila* diet with WO markedly increased the amount of ALA (from 40.47 to 148.0) and LA (from 1110.7 to 1705.0). OL levels increased from 683.5 to 846.0, while the levels of PA (from 655.65 to 724.48) and SA (from 939.85 to 966.56) did not change appreciably (levels calculated as μg analyte/mL diet ± SEM; data from three independent experiments). The unsaturated-to-saturated fatty acid ratio in WO is 9%, which is higher than the ratios found in peanut and macadamia oils [11,32].

Knowing the actual composition of the diet, including its supplements, is important for evaluating the effects it may have on the organism under both healthy and pathological conditions. Clinical and experimental studies have shown promising beneficial effects of PUFAs in various systemic pathologies, ranging from diabetes to cardiovascular disorders, as well as in several brain diseases [33]. This lipid fraction, naturally rich in omega-3 and omega-6 fatty acids, has demonstrated antioxidant properties, as evidenced by the modulation of glutathione reductase activity and the reduction in levels of oxidized proteins, DNA, and ROS damage in animals fed a diet enriched with omega-3 PUFAs. Due to their anti-inflammatory and antioxidant activity at the CNS level, PUFAs can attenuate oxidative stress and inflammation, representing a potential strategy against neurodegenerative diseases [34]. Similarly, another study reported that in transgenic mice, diets rich in DHA for 6 months were capable of normalizing brain lipid composition and decreasing susceptibility to oxidative damage without increasing α-synuclein levels [35]. These studies, like ours, demonstrate how PUFA supplementation can potentially prevent the onset of PD or slow its progression through an anti-apoptotic mechanism [36].

### 2.2. Effect of Treatment on Climbing Performance in Drosophila melanogaster

*Drosophila* exhibited a clear negative geotactic response, with approximately 90% of individuals reaching the upper section of the climbing apparatus within 5 s. This parameter was used to assess alterations in locomotor activity.

The dose of Rot (350 µM in the standard diet) was selected because it significantly decreased the percentage of individuals in the upper section (*p* < 0.001). This impairment in the negative geotaxis, reflected by the reduced ability to climb quickly in the apparatus, indicates a locomotor deficit induced by the mechanical insult. Additionally, a dose of 1.0 µL WO/mL in the supplemented diet was chosen for the present experiments from a range of 0.01 ÷ 10 µL/mL, as it represented an intermediate dose that did not affect the climbing parameters.

**Figure 3:**
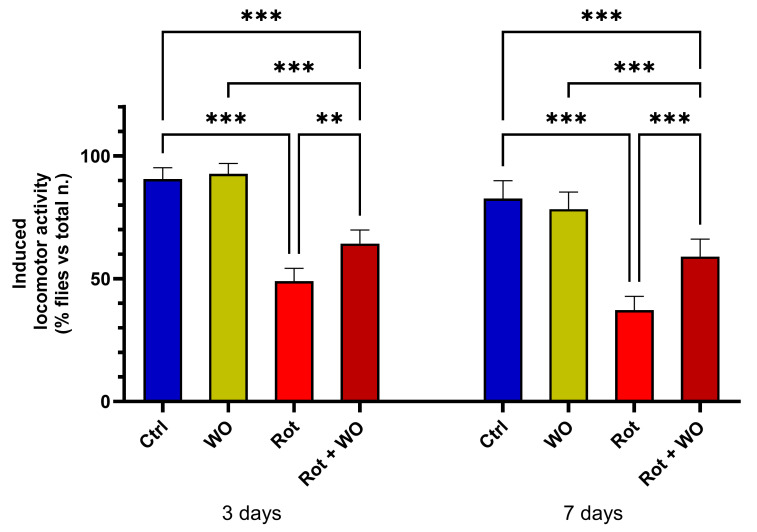
Effects of walnut oil (WO) and/or rotenone (Rot) in adult male of *Drosophila melanogaster* on negative geotaxis (3rd and 7th treatment day); bars indicate the percentage of flies that climb to the vial top within 5 sec (50 flies, five replicates, three independent groups, mean ± SEM). One-way ANOVA followed by Bonferroni’s multiple comparisons test: ** *p* < 0.01, *** *p* < 0.001.

Figure 3 shows the effects of WO and/or Rot on the climbing assay (upper section results).

After a 3-day treatment, WO alone did not induce significant changes in locomotor activity, while Rot treatment caused a marked deficit in motor performance compared to controls (*p* < 0.001). The combination of Rot and WO improved *Drosophila* performance in negative geotaxis, significantly increasing the percentage of flies in the upper section compared to those treated with Rot alone.

After 7 days of diet supplementation, the results were consistent with those observed after 3 days: WO (1 µL/mL) alone did significantly change the percentage of flies in the upper section. However, when combined with Rot, WO mitigated the reduction in the percentage of flies in the upper section caused by Rot alone, restoring these levels to values between those of the Rot-treated group and the control group.

Behavioral changes in *Drosophila melanogaster* resulting from chemically induced neurotoxicity have been extensively studied to assess the potential neuroprotective effects of various therapeutics [37]. As Rot has been shown to induce a significant change in negative geotaxis, we used a dosage of 350 µM to evaluate its effect on climbing behavior. This neurotoxin induces a PD-like phenotype in *Drosophila,* characterized by several behavioral changes, including a diminished innate negative geotaxis response due to locomotor defects. Rot generally injures dopaminergic neurons, leading to behavioral deficits, including impaired climbing ability, which can be measured using the negative geotaxis assay [38]. After both 3 and 7 days, flies exposed to the neurotoxin exhibited a significantly reduced climbing ability compared to the control group. However, the loss of negative geotaxis ability was significantly ameliorated when flies were co-treated with WO at the selected dosage.

The observed reduction in locomotor behavior and the deficit in negative geotaxis indicate that the PD model was successfully induced in flies specifically exposed to the neurotoxin rotenone.

Rot is one of the most widely used substances to induce a model of PD. This neurotoxin is a natural compound, originally isolated from the roots of plants belonging to the Leguminosae family. It has been used as an insecticide and as a toxin for controlling weed species in lake environments. Due to its high lipophilicity, Rot can freely cross the blood–brain barrier and accumulate in the CNS, where it selectively induces the degeneration of dopaminergic neurons in the *substantia nigra*.

Rot-induced mitochondrial deficiency can impair the locomotor system by causing energy deficits in the ambulatory and flight muscles, which are rich in mitochondria and have high energy demands. This bioenergetic crisis, resulting from the inhibition of electron transport chain complex I, adversely affects both locomotor and exploratory behaviors [39]. With its ability to reproduce the main pathological features of PD, it is a valuable tool for conducting neuroprotection studies [21,26].

### 2.3. Effect of Treatment on Longevity in Drosophila melanogaster

We assessed the lifespan of newly eclosed adult *Drosophila* maintained on medium supplemented with Rot (350 µM) and WO, and compared it with groups treated only with Rot or WO, as well as control groups. To evaluate lifespan, we recorded the time from eclosion until the death of all flies in each group, noting the number of flies that died every two days. The observation period lasted approximately 60 days, until all flies had died.

**Figure 4:**
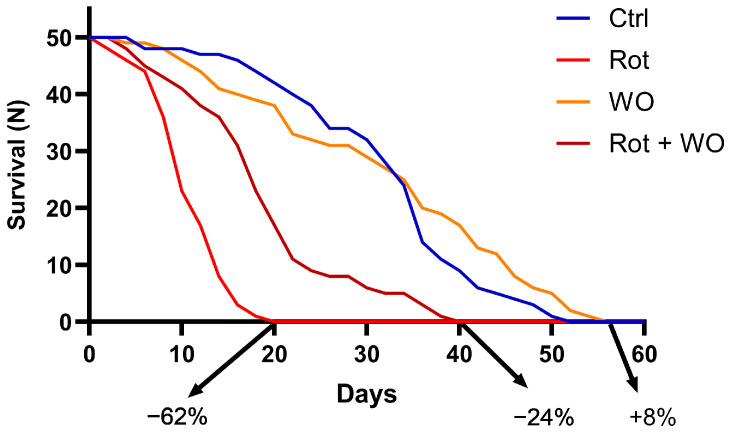
Effects of WO and/or Rot in adult male of *Drosophila melanogaster* on lifespan (50 flies; three separate experiments) after 60 days of treatment (the percentages in the figure represent the comparison with the control group survival rate). Survival curves were statistically analyzed by log rank test (Mantel Cox). SEM values were always below 10% and omitted from the graph for sake of clarity.

Longevity curves (Figure 4) showed an increase in mortality for the Rot alone group (−62%), while WO (1.0 μL/mL in the diet) induced a slight increase in survival compared to controls (+8%), marginally affecting the *Drosophila* lifespan. Co-administration of Rot and WO significantly reduced the mortality rate induced by Rot alone, resulting in intermediate values (−24% compared to control).

Treatment with Rot significantly reduced the median and maximum survival by 3.4-fold (10 days) and 2.9-fold (16 days), respectively, compared with control flies (34 and 46 days). WO slightly, but not significantly (*p* > 0.05), increased median (31 days) and maximum survival (50 days) compared to controls. When given in combination with Rot, WO counteracted Rot’s effects, significantly increasing the median and maximum survival by 1.8-fold (18 days) and 2.0-fold (32 days), respectively, compared to flies treated with Rot alone (*p* < 0.05).

In this study, flies exposed to Rot exhibited increased mortality and locomotor impairment, displaying a phenotype similar to PD. Previous studies with Rot have also shown high mortality and locomotor deficits in flies, characterizing the Rot-induced PD model in *Drosophila* [40,41].

Moreover, studies on different animal models of PD suggest that omega-3 PUFAs can improve motor and cognitive function by partially restoring dopaminergic neurotransmission [36]. In a *Drosophila* model of PD, adding the toxin paraquat to the nutrient medium severely impaired locomotor activity and reduced life expectancy. However, simultaneous intake of omega-3 fatty acids (EPA and DHA) with paraquat inhibited this reduction in life expectancy and improved locomotor activity [42].

Another study investigated the role of PUFA monoacylglycerides (MAGs) derived from marine sources as nonpharmacological dietary supplements to improve various pathological conditions and aging. The *Drosophila* short-lifespan model was sampled at five different ages. Supplementing the diet with MAG-EPA and MAG-DHA increased the lifespan of male *Drosophila* and modulated mitochondrial oxidative capacity and oxidative stress markers in thorax muscle [43].

In the present study we also found that flies treated with WO exhibited enhanced survival rates against Rot treatment in the co-exposure paradigm. This strongly suggests the neuroprotective role of WO in promoting survival pathways and mitigating locomotor impairments.

### 2.4. Modulatory Effect of WO against Rot-Induced Dopamine Depletion

**Figure 5:**
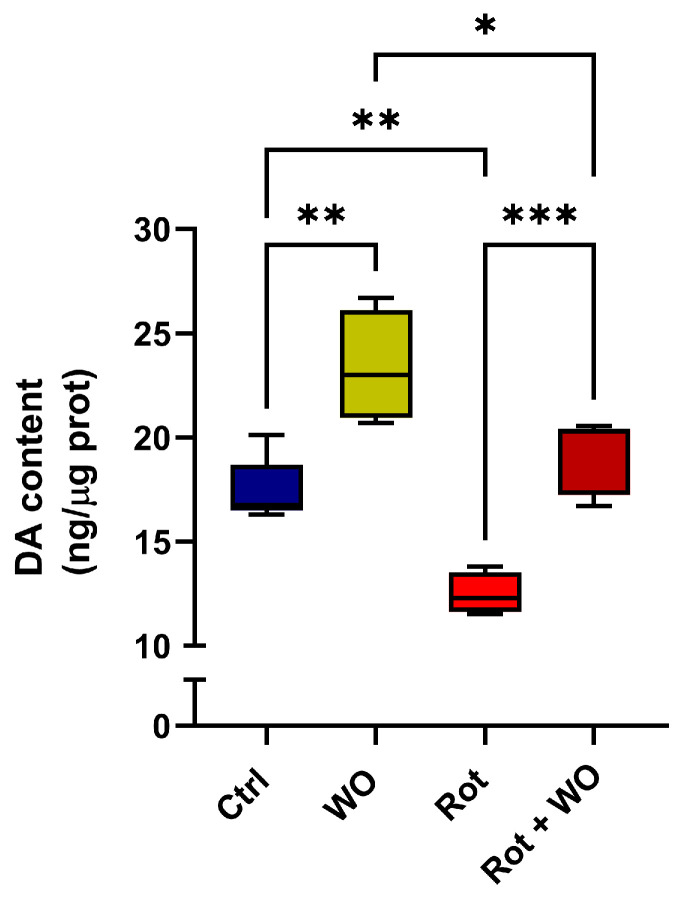
Dopamine (DA) content in *Drosophila* heads expressed as ng/μg protein, mean ± SEM of three independent experiments. One-way ANOVA followed by Bonferroni’s multiple comparisons test: * *p* < 0.05, ** *p* < 0.01, *** *p* < 0.001.

Flies treated solely with WO exhibited a 34% increase in DA levels in their heads. When treated with Rot alone, a significant 23% depletion in DA levels was observed compared to untreated control flies. Notably, the simultaneous administration of Rot and WO restored DA content in the fly heads to levels similar to those of the controls (Figure 5).

This suggests a role of the dopaminergic system in the amelioration of behavioral parameters (locomotion and survival) induced by Rot + WO, compared to Rot exposure alone, and indicates a correlation with changes in the fatty acid composition, particularly in PUFA content.

In humans, PD is a multifactorial disorder influenced by both genetic and environmental factors. Aging is the primary risk factor for the onset of the disease. The second most common risk factor is genetic. In 5–10% of cases, PD manifests as a Mendelian form with autosomal dominant or recessive inheritance. Significant progress in understanding the genetic contribution to the etiology of idiopathic PD has been made through genome-wide association studies (GWASs). These studies have investigated sex-related differences [44], single-country assessments [45], heterogeneity and therapeutic effects [46], and the correlation between PD risk genes and the disease’s onset [47].

Although *Drosophila* has a simpler nature due to its smaller genome and nervous system, consisting of approximately 200,000 neurons, it can still exhibit many of the high-ordered behavioral and molecular functions found in vertebrates, together with a high number of genes orthologous to human ones. Biogenic amines such as dopamine, serotonin, tyramine, and octopamine are known mediators of diverse physiological and behavioral functions in the fly [48].

Our results demonstrate the ability of WO to prevent the damage caused by this model, which may be related to the restored DA levels observed in the fly head. This restoration led to the prevention of locomotor impairments and an improvement in the survival of flies exposed to Rot + WO. An increasing number of studies have shown that Rot exposure produces behavioral and biochemical changes similar to those observed in PD and other neurological diseases [49,50]. Regarding the characterization of PD induced by Rot exposure, our results are consistent with other studies that have found DA depletion in *Drosophila melanogaster* [40,41]. DA depletion, along with reduced climbing ability and decreased survival rates, further validates the use of Rot as an in vivo model of PD. The toxicity caused by Rot can lead to signs and symptoms similar to those observed for PD. As previously highlighted, this is associated with mitochondrial dysfunction due to complex I inhibition, which decreases antioxidant levels in the electron transport chain, increases iron levels, and impairs dopaminergic neurons that may be more susceptible to oxidative stress than other neurons in the brain [51].

Pathologies associated with aging, such as PD, have been linked to the accumulation of mitochondrial mutations and the decline in mitochondrial function, as demonstrated through various mouse models and experimental strategies. Notably, several studies have established a causal relationship between mitochondrial DNA mutations and aging, with consequences including a reduced lifespan and increased susceptibility to diseases [52].

Therefore, we are among the first to verify that changes in the fatty acid profile in the head of flies in this PD model are correlated to DA modifications due to different treatments. WO not only reduced the depletion of DA caused by Rot, demonstrating its potential to restore DA levels and its neuroprotective effect, but also increased DA content when administered alone.

Several molecular pathways have been proposed for the development of PD, including oxidative stress and the generation of ROS. The increased incidence of age-associated diseases, such as PD, is indeed linked to the generation of free radicals and associated oxidative stress, which is enhanced in certain regions of the brain during aging [53]. Evidence suggests that Rot-induced neurotoxicity can be attributed to the specific sensitivity of dopaminergic neurons to radical species and oxidative damage [54]. Moreover, signs of oxidative damage have frequently been detected in dopaminergic neurons from PD patients and animal models, suggesting an implication of oxidative stress in this disease [54].

Recently, the antioxidant hypothesis of PD has become widely accepted, and the effectiveness of various neuroprotective agents in neurotoxin-based models has been demonstrated [55]. The inhibition of free radicals by antioxidant compounds offers protective effects against dopaminergic neurotoxicity. Fatty acids, particularly PUFAs, can easily penetrate and selectively accumulate in the fly brain, potentially reversing the increase in radical species in the fly head. These results suggest the potential of WO to attenuate oxidative damage while maintaining cellular homeostasis. 

Furthermore, a correlation between DA levels and some of the evaluated parameters can be observed. We found that a decrease in DA levels led to an increase in velocity in the negative geotaxis test and could also be correlated with a decrease in fly survival. Thus, a strong correlation exists between DA levels and both fly survival and behavioral parameters. These findings demonstrate the successful establishment of the Rot-induced model of PD in *Drosophila melanogaster*, corroborating other studies in the literature [40,41].

Our findings are consistent with other studies that have demonstrated the beneficial effects of dietary omega-3 PUFAs on toxin-induced neuronal degeneration in a PD mouse model [56]. In that research, mice were exposed to either a control diet or a high omega-3 PUFA diet from 2 to 12 months of age, and then treated with the neurotoxin 1-methyl-4-phenyl-1,2,3,6-tetrahydropyridine (MPTP). High omega-3 PUFA dietary consumption completely prevented the MPTP-induced decrease in tyrosine hydroxylase-labeled nigral cells and DA transporter mRNA levels in the *substantia nigra*, which are important markers of dopaminergic activity. Although omega-3 PUFA dietary treatment had no effect on striatal dopaminergic terminals, the high omega-3 PUFA diet protected against the MPTP-induced decrease in DA and its metabolite in the striatum. Taken together, these findings suggest that a high omega-3 PUFA dietary intake exerts neuroprotective actions in a mammalian model of Parkinsonism [56].

Similarly, several PD experimental models using striatal neuronal cultures, striatal slices, and mice were studied to assess the neuroprotective effects of DHA, the main omega-3 PUFA in the brain, administered in its triglyceride form (TAG-DHA). The beneficial effects of TAG-DHA were evaluated on neural viability following 6-hydroxydopamine-induced neurotoxicity, a well-established PD model [57].

In Rot-induced PD models, DHA demonstrated a neuroprotective effect in male rats pretreated with DHA for seven days and then administered Rot for eight days. The in vivo supply of DHA exerted neuroprotective effects, as evidenced by decreased dopaminergic neuron cell death. DHA also prevented the Rot-induced decrease in tubulin and synaptophysin expression in the rat striatum [58]. Given that DHA is metabolized in vivo to DHA epoxides and further hydroxylated to the corresponding diols, another study investigated the roles of these DHA metabolites in the beneficial effects of DHA supplementation using a similar Rot-induced rat model of PD. DHA supplementation in rats improved Rot-induced motor dysfunction. In addition, DHA reversed the Rot-induced decrease in tyrosine hydroxylase, reduced lipid peroxidation in the striatum, and increased mRNA expression of antioxidant genes in the striatum [59].

### 2.5. Quantitative Analysis of Fatty Acids in Drosophila Heads

Fatty acid levels in *Drosophila* heads were determined by LC-QO-MS analysis for confident compound identification. The derivatization of the target analytes with p-Bromophenacyl bromide (p-BPB) allowed for MS analyses in positive electrospray (ESI) mode, resolving the issue of poor ionization efficiency in negative ion mode, likely due to the moderate gas-phase acidity of the carboxylic group [60]. Moreover, the resulting phenacyl esters could be easily detected in the complex background of biological extracts due to the presence of a bromine atom, which produced two [M+H]^⊕^ ions with approximately equal intensity and separated by two mass units, according to the Br isotope pattern (^79^Br, 50.5% and ^81^Br, 49.5%).

The derivatization reaction led to a mass shift of approximately 197 a.m.u. (196.99) for the [M+H]^⊕^ ion; LC-QO-MS analyses of calibrators confirmed the theoretical *m*/*z* values calculated for the target analytes and showed the additional formation of a sodium adduct with high intensity for PA, OL, SA, and internal standard (IS) (Table 1). 

Identification of the target analytes and IS in *Drosophila* head extracts was performed based on retention time, mass accuracy, and isotopic fit values compared to those of the refence standards; the most intense ion was selected for quantification (quantifier ion) and calibration curves were generated by plotting the peak-area ratio of each analyte quantifier ion to that of the IS against the nominal analyte concentration. 

**Figure 6:**
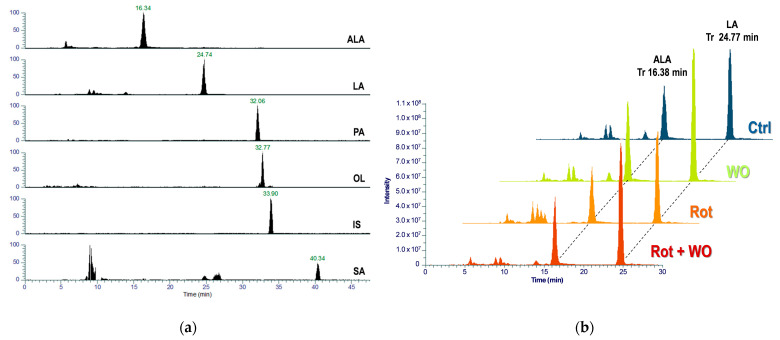
LC-QO-MS analysis of *Drosophila* head extracts: (**a**) representative chromatogram showing the traces for the selected quantifier MRM transitions in a head extract of *Drosophila* fed with the WO-enriched diet and (**b**) ALA and LA quantifier MRM transitions in the four animal groups.

Figure 6a presents a representative chromatogram showing the quantifier MRM transition traces for the target analytes and IS in a head extract of *Drosophila* fed with the WO-enriched diet, and Figure 6b shows superimposed chromatograms for ALA and LA quantifier MRM transitions across different animal groups.

**Figure 7:**
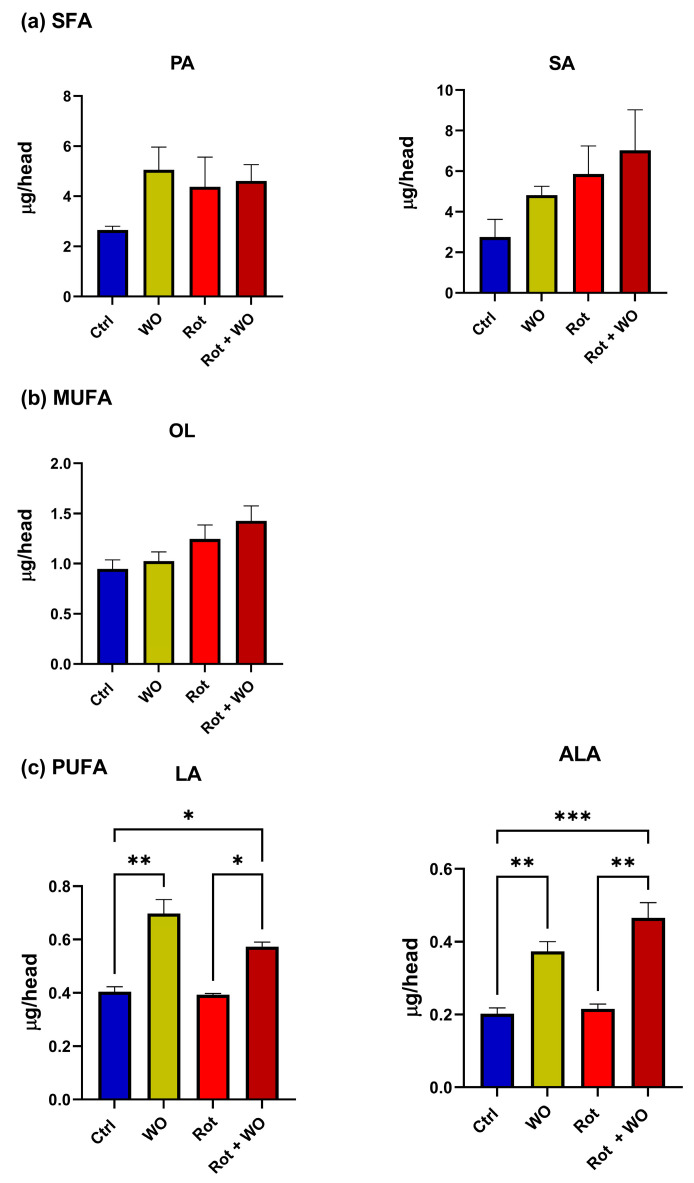
Fatty acid levels in *Drosophila* heads expressed as μg analyte/head: (**a**) saturated fatty acids (SFAs), (**b**) monounsaturated fatty acids (MUFAs), (**c**) polyunsaturated fatty acids (PUFAs). One-way ANOVA followed by Tukey’s multiple comparisons test: * *p* < 0.05, ** *p* < 0.01, *** *p* < 0.001.

Figure 7 shows the fatty acid levels found in the *Drosophila* head extracts. PA, SA, and OL levels did not change significantly after treatment with WO and/or Rot. Conversely, WO administration, either alone or in combination with Rot, significantly increased the levels of both ALA and LA compared to the control and Rot-only groups; moreover, their levels in the Rot + WO group were significantly greater compared to the control group.

In our study, we showed that dietary intake of omega-3 PUFAs can modify fatty acid concentrations in a *Drosophila* model of PD induced by Rot. Diets enriched with WO successfully increased ALA levels in the flies’ brain. PD, the second most prevalent neurodegenerative disorder, is characterized by α-synuclein aggregates, Lewy bodies, mitochondrial dysfunction, oxidative stress, and neuroinflammation. Recently, omega-3 PUFAs have gained attention as a potential preventive and therapeutic strategy for PD due to their antioxidant and anti-inflammatory properties. 

WO has been demonstrated to effectively scavenge free radicals, enhance total antioxidant capacity, and regulate the expression of related enzymes and genes (e.g., Klotho), thereby mitigating oxidative stress damage. Research shows that WO’s free radical scavenging ability surpasses that of common vegetable and nut oils, ranking as follows: WO > sesame oil > linseed oil > olive oil [61].

Research suggests that the protective benefits of omega-3 PUFAs on the brain may be due to their ability to restore omega-3 PUFA metabolism and integrate into cell membranes, impacting various brain functions [62]. The delivery of ALA to brain cells, along with its role in maintaining membrane flexibility, can trigger protective responses against oxidative stress, apoptosis, inflammation, and neurotrophic factors [62,63,64]. 

Fatty acids are essential in the diet of many animals throughout their lives. The mechanisms involved in the perception of and preferences for dietary saturated fatty acids (SFAs) and unsaturated fatty acids (UFAs) have been explored, using the model species *Drosophila melanogaster*, by Fougeron et al. [65]. Their studies revealed different preferences in larvae and adults, as determined by individual and group behavioral tests. Larvae preferred UFAs whereas adults generally preferred SFAs, laying more eggs and having a longer lifespan when consuming these substances. 

However, our research indicates that the lifespan of adult flies fed a diet supplemented with WO (1 µL/mL) for 7 days is slightly longer than that of controls, and under conditions of neurotoxin intoxication, the combination with WO, rich in PUFAs, is able to improve the fly survival rate compared to those exposed solely to the neurotoxin Rot.

Our findings are consistent with another study [66] that explored the need for PUFAs as essential nutrients for the normal functioning of the *Drosophila* nervous system. Flies raised on a diet low in PUFAs showed a significant reduction in head PUFA content (by 72%), while the same diet supplemented with WO increased both ALA and LA levels in the heads and simultaneously rescued the neurological disorders and defects in the flies’ visual system induced by a low PUFA diet. Moreover, adult flies fed a low PUFA diet enriched with ALA for 48 h were able to compensate for the lack of PUFAs in their heads and, in parallel, recovered the neurological functions.

In conclusion, PUFAs appear to be required in fly adulthood when there are defects induced by nutrient deficiency or toxins, in order to re-establish biochemical or behavioral balance.

A high-fat diet (HFD) has been shown to alter phenotypic and metabolic parameters in *Drosophila melanogaster*. However, the impact of fat quantity and quality is still under investigation. When fruit flies were fed with 12% various HFDs (butterfat, sunflower oil, olive oil, linseed oil, fish oil), their fatty acid profiles shifted according to the dietary fat qualities. In particular, ingestion of a PUFA-rich HFD (sunflower oil and linseed oil) induced a significant increase in PUFA content in both male and female *Drosophila,* along with a reduction in MUFAs. Additionally, ingestion of the HFD-linseed oil, which contains high levels of ALA, led to a substantial increase in ALA in both male and female flies. Furthermore, fat quality was found to determine the magnitude of the response to an HFD for traits such as lifespan, climbing activity, and fertility. In conclusion, the data from this study [67] indicate that not only fat content but also fat quality are crucial factors affecting life-history traits when applying an HFD in *Drosophila*.

## 3. Materials and Methods

### 3.1. Chemicals

Individual fatty acid standards were purchased from Merck (Milan, Italy) and stored at −20 °C: palmitic acid (PA; C16:0), stearic acid (SA; C18:0), oleic acid (OL; C18:1), linoleic acid (LA; C18:2), linolenic acid (ALA; C18:3), *cis*-5,8,11,14,17-eicosapentaenoic acid (EPA; C20:5), *cis*-4,7,10,13,16,19-docosahexaenoic acid (DHA; C22:6), arachidonic acid (ARA; C20:4), and eicosadienoic acid as internal standard (IS, C20). 2,4′-Dibromoacetophenone (p-Bromophenacyl bromide, pBPB), rotenone (Rot), acetone, 18-Crown-6, sodium hydroxide (NaOH), 2 M hydrochloric acid (HCl), hexane, and methanol (MeOH) HPLC grade were from Merck (Milan, Italy). Phenolphthalein solution 0.1% in ethanol 80% (PPT), chloroform, and acetonitrile (ACN) HPLC grade were purchased from VWR (Milan, Italy). HPLC-grade water (H_2_O) was obtained from a Milli-Q Water System (Millipore Corp., Burlington, MA, USA). Solvents were filtered through a 0.45 μm Millipore membrane filter and degassed before and during HPLC.

Jazz-Mix *Drosophila* food and *Kluyveromyces fragilis* yeast were supplied by Fisher Scientific (Milan, Italy) and VWR Chemicals (Milan, Italy), respectively.

A Novus Biologicals™ Universal Dopamine ELISA Kit was purchased from Fisher Scientific (Milan, Italy).

### 3.2. Walnut Oil

Walnut oil (WO) was obtained by cold pressing the *Juglans regia* L. walnuts grown in certified organic farming (production year: 2022; CiboCrudo, Milan, Italy) with the following declared composition: 9.1% SFA, 16.5% MUFA, and 69.9% PUFA. The oil did not undergo any refining or processing at high temperatures and was tested for the absence of heavy metals, aflatoxins, or mold.

### 3.3. Drosophila melanogaster Strain and Maintenance

White-eyed (w1118) strains of *Drosophila* flies were cultured on a standard diet composed by Jazz-Mix *Drosophila* food and *Kluyveromyces fragilis* yeast (190 mg and 16 mg, respectively, in 1.0 mL water). Groups of approximately 25–30 mating pairs were transferred into 50 mL culture vials containing 10 mL of standard diet and kept at 25 °C and 60% humidity on 12:12 photoperiod. After ten days, only newly enclosed male flies were collected over a period of three days and used in further experiments. 

### 3.4. Treatment of Drosophila melanogaster with WO and Rot

Newly eclosed male flies (1 to 3 days of age) were randomly separated into control and treated groups (about 50 flies/vial; 3 vials for each group) as follows: Ctrl group (standard diet), WO group (diet + WO), Rot group (diet + Rot 350 μM), and Rot + WO (diet + Rot 350 μM + WO). For WO enrichment, 10 μL WO was added to 10 mL standard diet; Rot molarity was considered as final in the standard diet. Newly eclosed groups (1 to 3 days old) of *Drosophila* males were transferred to vials with a fresh diet (control or treated), changed weekly, for longevity assay. Other groups were subjected to a climbing test after 3 and 7 days of treatment; thereafter, male flies were euthanized on ice and their heads were cut under a stereomicroscope with a scalpel for quantitative analysis of fatty acids and DA.

### 3.5. In Vivo Assays

#### 3.5.1. Short-Term Shock-Induced Locomotor Activity-Negative Geotaxis (Climbing)

Approximately 50 adult male flies were anesthetized and placed in an empty climbing vial (length, 12 cm; diameter, 3.0 cm) with the indication of three height ranges (top, medium, and bottom). After a brief recovery period, flies were gently tapped to the bottom of the vial and then allowed to climb for 5 sec. Flies were filmed to count the number of animals reaching each target line. The procedure was repeated 5 times for each vial (3 vials for each treatment group and control) and the reported data refer to the upper section (5 cm space from the bottom one), which is considered the most significant.

#### 3.5.2. Longevity Assay

Adult male flies, 1–3 days old, were grown up in the control or enriched/treated diet as described in Section 3.4 (about 50 flies/vial; 3 vials for each group), and then were transferred at least once a week into new vials with a fresh standard diet (control) or a diet enriched with WO and/or Rot as reported above. Mortality was recorded every two days for 60 days to track the longevity curves; the experiments were performed in triplicate and data were averaged across three determinations.

### 3.6. Sample Processing of Fatty Acid Analyses

#### 3.6.1. Lipid Extraction from WO and Drosophila Diet

Samples of 10 mg of WO were mixed with 2500 µL of acetone. Then, 180 µL aliquots were combined with 20 µL of IS solution (2.5 mg/mL, corresponding to 50 µg of IS in the sample) and dried completely using a SpeedVac vacuum concentrator (Fisher Scientific, Milan, Italy). 

For the *Drosophila* standard diet, 20 mg aliquots of Jazz-Mix *Drosophila* food or *Kluyveromyces fragilis* yeast were added with 20 μL IS solution (50 μg IS in the sample) and 1.0 mL chloroform: MeOH (2:1, *v*/*v*). Samples were sonicated for 10 × 10 s (Labsonic U, B. Braun, Melsungen, Germany), subjected to magnetic stirring at room temperature for 3 h, and centrifuged at 13,000 rpm for 5 min. The procedure to extract triglycerides was repeated twice and the collected supernatants were evaporated to dryness using a SpeedVac.

#### 3.6.2. Lipid Extraction from Drosophila Heads

Samples (3 pools of 30 heads for each treatment group and control) were added with 20 μL IS solution (50 μg IS in the sample) and 100 μL chloroform:MeOH (2:1, *v*/*v*) and left at 4 °C for 15 h to soften the exoskeleton. After micropestle-assisted homogenization (7 × 10 s) and sonication (10 × 10 s), samples were added with 400 μL chloroform:MeOH (2:1, *v*/*v*), subjected to sonication (15 min) and magnetic stirring (2 h at room temperature), and finally centrifuged at 13,000 rpm for 5 min to extract brain phospholipids. The procedure was repeated twice and the collected supernatants were evaporated to dryness using a SpeedVac.

#### 3.6.3. Lipid Saponification

The residues obtained in the processing procedures described above were added with 320 μL MeOH and 60 μL of 50% aqueous NaOH and stirred at 80 °C for 1.5 h. After cooling, samples were extracted with hexane (1.0 mL followed by 2 × 0.5 mL; total volume: 2.0 mL) to remove the unsaponifiable fraction. The aqueous phase was acidified with 2 M HCl and the undissociated target analytes were extracted with hexane (1.0 mL followed by 2 × 0.5 mL; total volume: 2.0 mL). The organic phases were combined and evaporated to dryness using a SpeedVac and the residues (free fatty acids) were stored at −20 °C until derivatization.

#### 3.6.4. Fatty Acid Derivatization

The obtained fatty acids were derivatized to their p-bromophenacyl esters [68] to introduce a chromophore group, which enabled detection for HPLC-UV analyses and also improved response in LC-ESI-MS, resolving the problem of poor ionization efficiency of fatty acids in negative ionization mode [69]

Briefly, samples were solubilized with 400 μL MeOH, added with 5 μL PPT solution, and treated with 0.1% methanolic KOH to phenolphthalein endpoint. The samples were evaporated to dryness using a SpeedVac and then added with 300 μL ACN and 100 μL of the derivatizing mixture (86.3 mM pBPB and 4.6 mM 18-crown-6, both in ACN). The reaction was conducted at 80 °C for 45 min under stirring; the samples were centrifuged at 13,000 rpm for 5 min and the collected supernatants were stored at −20 °C until HPLC analysis (injection volume: 10 μL).

### 3.7. HPLC Analyses of Fatty Acids

#### 3.7.1. Food Samples (HPLC-UV)

Analyses of food samples (WO, *Drosophila* food, and yeast) were performed on a JASCO chromatographic system composed of two PU-2080 Plus pumps, an HG-980-30 solvent mixing module, a thermostatted column compartment (CO-2067 Plus column oven), and a UV-2075 Plus detector set at 254 nm (Jasco Corporation, Tokyo, Japan). Manual injection was performed by a Rheodyne 7725i injection valve (IDEX Corporation, Rohnert Park, CA, USA) with a 10 μL loop. Samples were analyzed on a LiChroCART Purospher RP-18e column (125 × 4 mm; 5.0 μm) preceded by a Purospher RP-18e (4.0 × 4.0 mm; 5.0 μm) guard column (Merck kGaA, Darmstadt, Germany). Analyses were performed with a mobile phase of H_2_O (A) and ACN (B) under the following gradient conditions: 0.0–20.0 min, isocratic at 85% (B); 20.0–35.0 min, linear gradient from 85 to 95% (B); 35.0–45.0, isocratic at 95% (B); 45.0–46.0, linear gradient from 95 to 55% (B). A pre-equilibration period of 14.0 min was used between each run. The flow rate was 1.0 mL/min and the column temperature was 35 °C. The mobile phase was degassed during use by a solvent degasser mod. Degasys DG-1210 (Uniflows Co., Ltd., Tokyo, Japan). The chromatograph was controlled and data were evaluated by LC-NET 2/ADC Interface v. 2.0 and ChromNAV 2.0 Software, respectively (Jasco Corporation, Tokyo, Japan). The chromatographic conditions were optimized by analyzing standard solutions of the derivatized target analytes.

#### 3.7.2. Drosophila Heads (LC-QO-MS)

Analyses on head extracts was carried out with an Ultimate 3000 UHPLC system, consisting of a vacuum degasser, an HPG-3400RS high-pressure mixing biocompatible gradient pump, a WPS-3000 TRS thermostatted autosampler, a TCC-3000RS thermostatted column compartment, and a flow splitter with integrated flow control (Thermo Fisher Scientific, Waltham, MA, USA). The splitter allowed chromatographic separation using the same column, mobile phase gradient, and flow rate of HPLC-UV analyses; then, LC eluate was split prior to MS detection. Untargeted analyses were performed by a high-resolution Q Exactive™ Hybrid Quadrupole-Orbitrap™ mass spectrometer under the following operative parameters: scan range, *m*/*z* 200 ÷ 1000; acquisition, ESI positive mode; resolution, 70,000; spray voltage, +3.4 kV; inlet capillary temperature, 320 °C; auxiliary gas temperature, 290 °C; sheath gas and auxiliary gas flow rate, 37 and 28 arbitrary unit (AU), respectively; maximum ion injection time, 243 ms; automatic gain control (AGC), 3e6. The mass spectrometer was calibrated prior to each batch analysis using Thermo Scientific Pierce ESI calibration solutions to ensure the mass accuracy. The system was controlled and data were evaluated by Xcalibur software (version 4.6, Thermo Fisher Scientific, Waltham, MA, USA).

#### 3.7.3. Calibration Curves for Fatty Acid Analyses

Working solutions (n = 10) of the target analytes were prepared by diluting a stock solution with methanol to obtain concentrations in the range 0.5 ÷ 75 μg/mL for EPA, DHA, and ARA; 3.0 ÷ 450 μg/mL for ALA; 15.0 ÷ 2250 μg/mL for LA; and 13.0 ÷ 1950 μg/mL for PA, OL, and SA. The IS solution was prepared in methanol at a concentration of 2.5 mg/mL. For calibration samples, 180 μL aliquots of the working solutions were added with 20 μL IS solution (2.5 mg/mL; 50 μg IS in the sample) and evaporated to dryness using a SpeedVac. The obtained samples were derivatized as described above and analyzed in triplicate on three separate days by HPLC-UV to quantify fatty acid levels in food samples (WO, food, and yeast). Calibrators for LC-QO-MS analysis of *Drosophila* head extracts were prepared in the same way in the range 3.0 ÷ 69 μg/mL for ALA, LA, and OL and 15.0 ÷ 345 μg/mL for PA and SA. 

The peak-area ratio of each derivatized fatty acid to IS was plotted versus the analyte concentration and calibration curves were calculated using the method of least squares using a weighed (1/x) linear regression model. All calibration curves exhibited R^2^ values > 0.991 for both HPLC-UV and LC-QO-MS analyses. The lower limit of quantitation (LLOQ) value for each analyte was estimated from the corresponding calibration plot as 10σ/S, where σ and S are the standard deviation and the slope of the regression line; all obtained values were below the lowest calibrators for both HPLC-UV and LC-QO-MS procedures.

### 3.8. Dopamine Analysis in Drosophila Heads

Quantitative analysis of DA in fly heads was performed using the ELISA enzyme immunoassay technique following the protocol reported by the manufacturer. Data were acquired on a microplate reader (Multiscan FC, Thermo Scientific, Waltham, MA, USA) by measuring the absorbance at λ = 450 nm. DA levels were determined from a standard curve generated with calibrators included in the kit (calibration range: 31.25 ÷ 2000 pg/mL; assay sensitivity: 18.75 pg/mL). Data, expressed as ng/μg protein, are presented as mean ± SEM from three independent experiments; protein amount was estimated using the Bradford method [70]. 

### 3.9. Statistical Analysis

The Graph-Pad Prism program v. 10.2.3 (GraphPad Software Inc., San Diego, CA, USA) was used for data analysis and graphic presentation. Each experiment was performed at least three times, and all values are presented as mean ± standard error of the mean (SEM). 

One-way analysis of variance (ANOVA) was used to compare differences among groups, followed by Bonferroni multiple comparisons test for climbing and DA data and by Tukey’s multiple comparisons test for the other data. The Kaplan–Meier method was used to compare the survival curves of *Drosophila melanogaster* and the survival differences were tested for statistical significance using the log rank test (Mantel Cox).

The authors prepared references using Zotero (version 6.0.36) as bibliography software.

## 4. Conclusions

In conclusion, we chose WO because it is particularly rich in PUFAs, which constitute 70% of its total fatty acid content.

We investigated the neuroprotective effects of WO using *Drosophila melanogaster* as a model organism. Our study involved four groups of flies: one fed a WO-enriched diet, another treated with Rot, a third group given a combination of both WO and Rot, and a control group. The results demonstrated that WO supplementation improved locomotor activity and reduced mortality in flies exposed to Rot. LC-MS analysis revealed significant increases in ALA and LA levels in the heads of flies fed a WO-enriched diet, as well as in those treated with both WO and Rot. Notably, flies fed WO exhibited elevated DA levels in the brain, while Rot treatment significantly depleted DA. However, when WO was combined with Rot, DA levels were maintained at levels comparable to the controls. These findings suggest that the increased intake of PUFAs, particularly ALA, from the WO-enriched diet plays a neuroprotective role against Rot-induced neurotoxicity. The enhancement of both behavioral and biochemical parameters in WO-fed *Drosophila* indicates that the dopaminergic system may mediate these protective effects. Finally, this study highlights the potential of WO supplementation as a promising nutritional strategy to prevent or slow the progression of neurodegenerative diseases, including PD.

## Figures and Tables

**Table 1:** Theoretical and experimental *m*/*z* values for LC-QO-MS analyses of the target analytes and IS as phenacyl esters in the calibration samples; (a) ^79^Br and (b) ^81^Br; * quantifier ion.

	Fatty Acid Formula	Phenacyl Ester Formula	Retention Time (min)	[M+H]^⊕^, *m*/*z* Theoretical	[M+H]^⊕^, *m*/*z* Experimental	[M+Na]^⊕^, *m*/*z* Experimental
ALA	C_18_H_30_O_2_	C_26_H_35_BrO_3_	16.38	(a) 475.1842 (b) 477.1822	(a) 475.1853 * (b) 477.1826	
LA	C_18_H_32_O_2_	C_26_H_37_BrO_3_	24.77	(a) 477.1999 (b) 479.1978	(a) 477.2007 * (b) 479.1982	
PA	C_16_H_32_O_2_	C_24_H_37_BrO_3_	32.09	(a) 453.1999 (b) 455.1978	(a) 453.1996 (b) 455.1971	(a) 475.1823 * (b) 477.1801
OL	C_18_H_34_O_2_	C_26_H_39_BrO_3_	32.80	(a) 479.2155 (b) 481.2135	(a) 479.2163 (b) 481.2141	(a) 501.1982 * (b) 503.1960
IS	C_20_H_36_O_2_	C_28_H_41_BrO_3_	33.93	(a) 505.2312 (b) 507.2291	(a) 505.2316 (b) 507.2293	(a) 527.2146 * (b) 529.2123
SA	C_18_H_36_O_2_	C_26_H_41_BrO_3_	40.38	(a) 481.2312 (b) 483.2291	(a) 481.2319 (b) 483.2296	(a) 503.2138 * (b) 505.2117

## Data Availability

The data presented in this study are available on request from the corresponding authors.
